# Supplementary material for: WordCommentsAnalyzer: A windows software tool for qualitative research
Source: F1000Res. 2018 Sep 4;7:536. Originally published 2018 May 3. [Version 2] doi: 10.12688/f1000research.14819.2 (PMC6137411; doi:10.12688/f1000research.14819.2)
Supplement: Supplementary file 3 [file f1000research-7-17614-s0002.tgz › b2a79496-4ada-47c4-9682-a99ce867836d.zip!/Eight Iranian Health Journals/Yafteh _22.docx]

© Yafteh  . This work is licensed under CC BY 4.0 (https://creativecommons.org/licenses/by/4.0/).

Volume & Issue: دوره 15 - شماره 1

Title: ارزیابی وضع تغذیه بیماران بستری در بخش ارتوپدی مرکز آموزشی درمانی شهدای عشایر خرم‌آباد در حین بستری و ترخیص

http://yafte.lums.ac.ir/article-1-1184-fa.html

مقدمه: سوء تغذیه یک عامل مهم در افزایش مدت بستری در بیمارستان، عوارض ناشی از بیماری ها، هزینه های درمان و افزایش میزان مرگ و میر می باشد. این مطالعه به منظور بررسی وضعیت تغذیه ای، حین بستری و زمان ترخیص بیماران بستری در بخش ارتوپدی بیمارستان شهدای عشایر در سال 1389 انجام شده است. بحث و نتیجه‌گیری: بر اساس یافته‌های این مطالعه می‌توان گفت ارتباط آماری معناداری بین طول مدت بستری بیماران در بیمارستان و بروز و حتی شدت سوء تغذیه وجود دارد. یافته‌ها: 7/12 درصد بیماران سوء تغذیه شدید، 4/7 درصد سوء تغذیه متوسط و 9/79 درصد سوء تغذیه خفیف داشتند. بین مدت بستری، جنس، کاهش وزن یک ماهه اخیر، سابقه جراحی قبلی با شدت سوء تفذیه ارتباط معنا دار آماری وجود داشت (05/0< P). بین سطح سواد، سن، وضعیت اقتصادی، بیماری زمینه ای، کاهش وزن شش ماه اخیر با شدت سوء تغذیه ارتباطی وجود نداشت (05/0> P). مواد و روش‌ها: در این مطالعه توصیفی- مقطعی، 283 بیمار بالای 18 سال بخش ارتوپدی مورد بررسی قرار گرفتند. پرسشنامه ای که از قبل طراحی شده و حاوی 15 متغیر کمی و کیفی بود برای بیماران در دو مرحله تکمیل شد (حین بستری و حین ترخیص) وزن و قد بیماران حین بستری و در زمان ترخیص اندازه گیری و در پرسشنامه مکتوب گردید. سپس با استفاده از نرم افزار آماری SPSS داده ها مورد تجزیه و تحلیل قرار گرفت.

Title: بررسی علل شکایات بیماران از پزشکان و دندان پزشکان در مراجعه کنندگان به معاونت درمان دانشگاه علوم پزشکی لرستان طی سال های 90-87

http://yafte.lums.ac.ir/article-1-1183-fa.html

مقدمه: در سال‌های اخیر تعداد شکایات از پزشکان و دندان‌پزشکان افزایش داشته است. شکایت از پزشکان سبب اتلاف انرژی و وقت در این گروه می‌شود. این مطالعه با هدف تعیین علل شکایات بیماران از پزشکان و دندان‌پزشکان مراکز درمانی در استان لرستان از سال 1387 تا 1390 انجام شد. بحث و نتیجه‌گیری: با توجه به علل شکایات انجام گرفته به نظر می رسد که با برقراری رابطه نزدیک‌تر با بیمار و ارائه توضیح بیشتر در مورد شرایط و عوارض اقدامات درمانی می توان از شکل گیری بسیاری از شکایات پیشگیری نمود. یافته‌ها: در این محدوده زمانی 328 شکایت ثبت گردیده بود که 168 پرونده مورد بررسی قرار گرفت. میانگین سن شاکیان 2/11±34 سال بود. 112 نفر از شاکیان زن بودند. بیشترین شکایات صورت گرفته از دندان پزشکان، متخصصین زنان و ماماها و متخصصین ارتوپدی بود. 25% از شکایت‌ها ناشی از صدمات جسمانی و عوارض جانبی درمان و 6/22% ناشی از سهل انگاری، کوتاهی و بی توجهی پزشک بودند. مواد و روش‌ها: این مطالعه توصیفی مقطعی در سال 1390 در شورای پژوهشی دانشگاه علوم پزشکی لرستان تصویب گردید. در پایان سال 1390، پرونده های شکایات ارجاع شده به معاونت درمان دانشگاه از سال 87 تا پایان نیمه اول سال 90 مورد بررسی قرار گرفت. پرونده هایی که حاوی اطلاعات مورد نیاز نبودند از مطالعه خارج شدند. داده های استخراج شده وارد نرم افزار SPSS شدند. در نهایت نتایج مطالعه به صورت درصد فراوانی و میانگین محاسبه و ارائه گردید. در این طرح هیچ گونه تعارض منافعی وجود نداشت.

Title: اثر اعتیاد به مواد مخدر بر روی عملکرد تیروئید

http://yafte.lums.ac.ir/article-1-1185-fa.html

مقدمه: اگرچه تصور می شود که مصرف مواد مخدر سبب تغییر در عملکرد تیروئید می شود ولی مکانیسم دقیق آن هنوز به خوبی مشخص نشده است. بنابراین، در ادامه یک مطالعه مقدماتی،هدف این مطالعه تکمیلی بررسی تأثیر اعتیاد به مواد مخدر بر روی عملکرد تیروئید با اندازه گیری TBG بود. مواد و روش ها: این تحقیق به صورت یک مطالعه مورد-شاهد بر روی 106 نفر معتاد به مواد مخدر و 106 نفر فرد سالم در تهران صورت گرفت. از نمونه های سرم این افراد برای اندازه گیری TBG استفاده شد. یافته ها: میزان TBG در گروه مورد به صورت معنی داری در مقایسه با گروه شاهد افزایش داشت(005/0

Title: بررسی علل شکایت مطرح شده به سازمان نظام پزشکی شهر خرم آباد

http://yafte.lums.ac.ir/article-1-1186-fa.html

مقدمه: شکایت از پزشکان یکی از مشکلاتی است که ممکن است هر پزشکی در طول دوره طبابت با آن روبرو شود. این مسأله سبب اتلاف انرژی و وقت و نیز ایجاد استرس در پزشکان می شود. طبق آمارهای موجود شکایات در ایران رو به افزایش است. شناخت این علل می‌تواند در پیشگیری از بروز شکایت مؤثر باشد. این طرح با هدف تعیین علل شکایت مطرح شده به سازمان نظام پزشکی شهر خرم‌آباد از سال 1385 تا 1390 انجام گرفت مواد و روش‌ها: مطالعه حاضر از نوع توصیفی- مقطعی و گذشته‌نگر است. برای انجام این مطالعه تمام شکایاتی که از سال 1385 تا سال 1390 در سازمان نظام پزشکی خرم‌آباد مطرح شده بود، مورد بررسی قرار گرفتند. اطلاعات مورد نظر از پرونده‌ها به پرسشنامه منتقل گردید. سپس داده‌های جمع‌آوری شده در نرم‌افزار آماری وارد شده و با آزمون‌های آماری مورد تجزیه و تحلیل قرار گرفتند. بحث و نتیجه‌گیری: رعایت اخلاق پزشکی و تعهد حرفه‌ای، برقراری ارتباط مناسب پزشک و کادر درمان با بیمار و همراهان او، توضیح کامل درمان و عوارض احتمالی آن، عدم بزرگ نمایی و اغراق در نتایج درمانی، مطالعه مستمر و به روز کردن دانش پزشکی، مهارت لازم و تجربه کافی پزشک، استفاده نکردن از روش هایی که دوره های آموزشی آن را نگذارنده باشند، انتخاب صحیح بیمار و اخذ رضایت آگاهانه و برائت ممکن است به کاهش شکایت از پزشکان منجر شود. یافته‌ها: در این مطالعه 260 مورد شکایت بررسی شد. بیشترین شکایات مطرح شده از بیمارستان های دولتی بود (8/68 درصد) . بیشترین علت شکایات مطرح شده مربوط به بی‌احتیاطی (4/55 درصد) و کمترین آن مربوط به عدم رعایت نظامات دولتی (21 درصد) بود. ازتعداد 260 مورد مطرح شده در هیئت بدوی 80 مورد (8/30 درصد) قصور و 180 مورد (2/69 درصد) به عنوان عدم قصور تشخیص داده شد. بر اساس نتیجه هیئت تجدیدنظر از کل 260 مورد، 61 مورد (5/23 درصد) قصور و 199 مورد (5/76 درصد) عدم قصور تشخیص داده شد.

Title: جداسازی باکتریهای بومی تولید کننده آنزیم کلسترول اکسیداز از خاک، آب ، پساب کارخانه های چرم و پوست ، صابون سازی و فراورده های لبنی

http://yafte.lums.ac.ir/article-1-1187-fa.html

بحث و نتیجه‌گیری: در این مطالعه، باکتریهای بومی مولد کلسترول اکسیداز جدا گردید. این دو باکتری بواسطه خصوصیات مورفولوژیکی، بیوشیمیایی و روشهای تائید مولکولی جزء گونه Rhodococcus طبقه بندی گردید. مواد و روش‌ها: تعداد 187 نمونه از پساب کارخانه چرم وپوست و صابون سازی ، خاک ، لبنیات و آب های راکد جمع آوری گردید. پس از کشت نمونه ها، باکتریهای رشد یافته با تست های میکروبی و بیوشیمیایی تعیین هویت گردید. برای تایید فعالیت کلنی های مولد آنزیم کلسترول اکسیداز، روشهای رنگ سنجی و قهوه ای نمودن محیط کشت استفاده شد. از تکنیک 16s rRNA PCR برای تایید قطعی سویه های بدست آمده استفاده شد. یافته‌ها: از 187 نمونه ،تنها 2 باکتری از خاک جداسازی گردید و نتایج نشان داد که متعلق به گونه رودوکوکوس می باشند . همچنین در آزمایش قهوه ای نمودن و رنگ سنجی، فعالیت کلسترول اکسیدازی هر دو باکتری تایید شد. هویت مولکولی سویه های مزبور با تعیین توالی ژن 16s rRNA تایید شد. مقدمه: کلسترول اکسیداز آنزیمی است که اکسیداسیون کلسترول را همراه با احیای اکسیژن مولکولی به پراکسیدهیدروژن انجام می دهد . این آنزیم توسط برخی میکروارگانیسم های بیماریزا و غیر بیماریزا تولید می شود و ازمهمترین آنزیم های تجاری دنیاست که کاربردهای وسیعی در صنایع مختلف پیدا کرده است. هدف از این مطالعه جداسازی باکتریهای بومی مولد کلسترول اکسیداز و تعیین هویت آنها با استفاده از روشهای میکروبی، بیوشیمیایی و ژنتیکی می باشد.

Title: بررسی مرگ و میر ناشی از مسمومیت ها طی سال های 1386 لغایت 1390 در بیماران بستری در بیمارستان شهدای عشایر شهرستان خرم آباد

http://yafte.lums.ac.ir/article-1-1188-fa.html

مقدمه: یکی از شایعترین علل مرگ و میر در تمام دنیا ناشی از مسمومیت هاست. تحقیق و بررسی علل مرگ ومیر ناشی از مسمومیت ها در اتخاذ تصمیمات و بهبود استانداردهای لازم برای پیشگیری از حوادث ناگوار، نقش مهمی را ایفا می کند. لذا برای شناخت بهتر علل و عوارض منتج به فوت بیماران مسموم، تصمیم به بررسی مرگ ومیر ناشی از مسمومیت ها طی سالهای 1386 لغایت 1390 در بیماران بستری در بیمارستان شهدای عشایر شهرستان خرم آباد گرفتیم. بحث و نتیجه‌گیری: در استان لرستان مسمومیت با سموم دفع آفات مخصوصاً قرص برنج (فسفیدآلومینیوم) و اپیوم ها و مرگ و میر ناشی از آنها بیشتر از سایر داروها و سموم می باشد. یافته‌ها: از میان 13090 بیمار دچار مسمومیت مراجعه کننده به این مرکز، 124 نفر بر اثر شدت عوارض حاصله فوت کرده بودند که 9/58% از آنان مرد بودند. اکثریت بیماران مجرد (6/51%) ، دارای دیپلم (5/43%) و ساکن شهر (4/77%) بودند. 4/73% بیماران فوت شده به طور عمدی و خودآزارانه اقدام به خودکشی کرده بودند. بیشترین سموم مصرفی به ترتیب سموم دفع آفات (2/53%)، اپیوییدها (8/21%) و داروهای مسکن-هایپنوتیک (5/10%) بودند. مواد و روش‌ها: این مطالعه ی توصیفی - تحلیلی به صورت مقطعی روی بیماران فوتی بستری شده ناشی از مسمومیت در بیمارستان شهدای عشایر طی سالهای 1386 لغایت 1390 صورت گرفته و اطلاعات مورد نظر در قالب پرسشنامه گرد آوری و با روشهای آمار توصیفی نظیر جداول توزیع فراوانی، جداول توافقی، میانگین، انحراف معیار و نسبت مورد تجزیه و تحلیل قرار گرفت.

Title: بررسی ارتباط متغیرهای اقلیمی و بروز حملات آسم منجر به بستری در بیمارستان کودکان شهر خرم‌آباد از سال 1379 لغایت 1387

http://yafte.lums.ac.ir/article-1-1189-fa.html

مقدمه: آسم یکی از شایع ترین بیماری های مزمن دوران کودکی و یکی از عوامل عمده بستری بیمارستانی در این سنین می باشد. از علل عمده مرگ و میر فزاینده ناشی از آسم، ضعف در تشخیص و دست کم گرفتن شدت بیماری تأخیر در ارجاع و درمان نامناسب را می توان نام برد. بنا بر این تشخیص زودرس کودکان در معرض خطر برای پذیرش فوری در واحد مراقبت های ویژه و همچنین شناسایی عوامل محیطی مؤثر در بروز و تشدید این بیماری سهم مهمی در مدیریت بیماری آسم بازی می کند. بحث و نتیجه‌گیری: مقایسه‌ نتایج حاصل از این پژوهش با سایر تحقیقاتی که در رابطه با تأثیر شرایط اقلیمی بر بیماری آسم انجام شده بیانگر این مسئله است که متغیرهای مؤثر شرایط حرارتی محیط همچون حداقل مطلق دما و روزهای توام با یخبندان همبستگی خوبی با حملات آسم داشته‌اند. اما نکته حائز اهمیت نوع متغیرهاست که در تحقیقات گذشته ارتباطی بین ساعات آفتابی و تعداد روزهای یخبندان با بیماری مورد بحث نشان داده نشده بود. یافته‌ها: در گام اول متغیر معدل ماهانه ساعات آفتابی با ضریب همبستگی 15/37 درصد و مقدار 058/0 =p به مدل وارد و صحت متغیر تأیید گردید . در گام دوم متغیر حداقل مطلق دما با فرض ثابت بودن متغیر اول نسبت به متغیر وابسته با ضریب همبستگی 5/31 درصد به مدل رگرسیون وارد شد . در گام سوم نیز متغیر تعداد روزهای یخبندان با ضریب همبستگی 5/9 درصد به مدل وارد و ضریب همبستگی چندگانه به 15/78 درصد رسید. نتایج نشان می دهد که از میان متغیرهای اقلیمی، معدل ماهانه ساعات آفتابی با رابطه معکوس، حداقل مطلق دما و معدل ماهانه تعداد روزهای یخبندان با رابطه مستقیم به ترتیب بیشترین همبستگی را با حملات آسم کودکان داشته اند. مواد و روش‌ها: در این پژوهش ارتباط بین متغیر های اقلیمی چون دما، تبخیر، رطوبت نسبی، ساعات آفتابی، تعداد روز های یخبندان و سمت و سرعت باد با تعداد دفعات حملات آسم کودکان شهر خرم آباد که منجر به بستری شدن آنها در بیمارستان آموزشی درمانی شهید آیت الله مدنی (تنها بیمارستان کودکان) این شهر شده است مورد بررسی قرار گرفته است . به منظور بررسی و شناسایی متغیرهای اقلیمی مؤثر در بروز حملات آسم کودکان از شیوه آماری رگرسیون چندگانه به روش گام به گام استفاده شده است. در این روش تعداد 10 متغیر اقلیمی مستقل با متغیر وابسته حملات آسم کودکان دریک دوره 9 ساله از سال 1379 تا سال 1387 مورد بررسی قرار گرفته و طی سه گام سه متغیر اقلیمی مؤثر در برآورد تعداد مراجعات حملات آسم کودکان شناسایی شده اند.

Title: اثرات مصرف شیر سویا بر فاکتورهای التهابی، انعقادی و استرس اکسیداتیو در بیماران دیابتی نوع دو مبتلا به نفروپاتی

http://yafte.lums.ac.ir/article-1-1190-fa.html

مقدمه: نفروپاتی یکی از عوارض معمول دیابت است و از علل عمده ناتوانی و مرگ در این بیماران محسوب می شود. هدف این مطالعه بررسی اثرات مصرف شیر سویا بر التهاب، انعقاد و استرس اکسیداتیو در بیماران دیابتی نوع دومبتلا به نفروپاتی است. مواد و روش‌ها: این کارآزمایی بالینی متقاطع تصادفی کنترل شده، بر روی بیماران دیابتی نوع دو مبتلا به نفروپاتی انجام شد. این مطالعه دو مرحله مداخله هر کدام به مدت چهار هفته و یک مرحله آبگیری به مدت دو هفته داشت. بیماران به صورت تصادفی یک رژیم غذایی حاوی شیر سویا یا رژیم غذایی حاوی شیر گاو را به مدت چهار هفته مصرف نمودند. بحث و نتیجه‌گیری: مصرف شیر سویا به مدت چهار هفته می تواند موجب کاهش سطح D-dimer در بیماران دیابتی نوع دو مبتلا به نفروپاتی شود. یافته‌ها: مصرف شیر سویا در مقایسه با مصرف شیر گاو در بیماران نفروپاتی دیابتی موجب کاهش معنی داری در سطح D-dimer، شاخص تشکیل و تخریب فیبرین، گردید. این معنی داری حتی پس از تعدیل عامل مخدوشگر (میزان دریافت کربوهیدرات) باقی ماند. مصرف شیر سویا اثر معنی داری بر سطح فاکتورهای التهابی و استرس اکسیداتیو مالون نداشت.

Title: بررسی ویژگی‌های روان‌سنجی و ساختار عاملی مقیاس بینش شناختی بک(BCIS) در نمونه غیر بالینی ایرانی

http://yafte.lums.ac.ir/article-1-1191-fa.html

مقدمه: بینش مفهومی پیچیده و چند بعدی است. سنجش‌های بالینی بینش، عمدتاً بر آگاهی بیماران از بیماری روانی و نیاز آنها به درمان متمرکز شده است. رویکردهای نوین بر فرآیندهای شناختی نظیر ارزیابی مجدد بیماران از تجارب غیرعادی‌شان و سوء تفسیرهای آنها تمرکز کرده‌اند. هدف پژوهش حاضر بررسی ساختار عاملی نقیاس بینش شناختی بک، در نمونه غیربالینی ایرانی است. مواد و روش‌ها: به این منظور، 476 نفر از دانشجویان دانشگاه‌های مختلف، مقیاس بینش شناختی بک (BCIS) و پرسشنامه خودآگاهی (SCS) را تکمیل کردند. داده‌های گردآوری شده به وسیله نرم‌افزار لیزرل و SPSS تجزیه و تحلیل شدند. بحث و نتیجه‌گیری: یافته‌ها پیشنهاد می‌کنند که مقیاس بینش شناختی بک (BCIS) دارای ثبات ساختاری لازم در جمعیت غیربالینی ایرانی می‌باشد و می‌تواند به عنوان یک ابزار سودمند، برای مقاصد پژوهشی مورد استفاده قرار بگیرد. یافته‌ها: یافته‌ها نشان دادند که دو مؤلفه اساسی خود-اندیشمندی و اطمینان به خود که توسط بک، باروچ، بالتر، استیر و وارمان(2004) مطرح شده بودند، در پژوهش حاضر نیز به عنوان عامل‌های مستقل به دست آمدند.

Title: تاثیر بازی‌های دبستانی بر رشد مهارت جابه‌جایی دانش‌آموزان پسر مبتلا به اختلال بیش‌فعال/ نقص توجه

http://yafte.lums.ac.ir/article-1-1192-fa.html

مقدمه: کودکان بیش فعال/نقص توجه دارای عملکرد حرکتی پایین‌تر از کودکان عادی هستند بنابراین هدف از پژوهش حاضر، بررسی تاثیر بازی‌های دبستانی بر رشد مهارت‌های جابه‌جایی دانش‌آموزان پسر مبتلا به اختلال بیش‌فعال/نقص‌ توجه پایه اول تا سوم ابتدایی شهرستان خرم‌آباد است. بحث و نتیجه‌گیری: لذا با توجه به یافته‌های این پژوهش مبنی بر اینکه بازی‌های دبستانی منجر به بهبود مهارت‌های جابه‌جایی دانش‌آموزان بیش‌فعال شده است پیشنهاد می‌شود این بازی‌ها در برنامه ی تربیت بدنی مدارس ابتدایی گنجانده شوند. یافته‌ها: یافته‌ها حاکی از آن است که داده‌ها نرمال بوده و بازی‌های دبستانی باعث بهبود مهارت‌های جابه‌جایی دانش‌آموزان بیش‌فعال/نقص‌توجه گروه آزمایش شده است همچنین گروه آزمایش نسبت به گروه کنترل در مهارت‌های جابه‌جایی دارای برتری می‌باشد. مواد و روش‌ها: بدین منظور از بین40 دانش‌آموز مبتلا به اختلال بیش فعالی تعداد 20 دانش آموز پسر انتخاب شده و براساس سن و نتایج پیش آزمون(سن 64/0=P، جابه‌جایی85/0=P) که به وسیله‌ی آزمون رشد حرکتی اولریخ انجام شد به دو گروه کنترل و آزمایش تقسیم شدند. سپس گروه آزمایش به مدت 18 جلسه و هر جلسه 45 دقیقه تحت تاثیر بازی‌های دبستانی قرار گرفت. در پایان مهارت‌های جابه‌جایی توسط ابزار اولریخ ارزیابی شد. که برای بررسی نرمال بودن داده‌ها از آزمون کلموگراف اسمیرنوف و برای تحلیل داده‌ها از آزمونT همبسته و برای مقایسه‌ داده‌ها در پیش‌آزمون و پس‌آزمون از آزمون T مستقل استفاده گردید، سطح معناداری در این تحقیق 05/0 P< می باشد.

Title: تاثیر تمرین مقاومتی با شدت کم در مقابل تمرین مقاومتی با شدت زیاد بر ساختار و عملکرد بطن چپ پسران نوجوان سالم با استفاده از اکوکاردیوگرافی

http://yafte.lums.ac.ir/article-1-1193-fa.html

مقدمه: مطالعه در زمینه تأثیر پروتکل‌های مختلف تمرین مقاومتی بر بطن چپ پسران نوجوان محدودیت دارد. هدف از مطالعه حاضر، تعیین تأثیر تمرین مقاومتی با شدت کم در مقابل تمرین مقاومتی با شدت زیاد بر ساختار و عملکرد بطن چپ پسران نوجوان سالم بوسیله اکوکاردیوگرافی بود. مواد و روش‌ها: بیست و چهار آزمودنی داوطلب 15 تا 18 ساله به طور تصادفی در سه گروه هشت نفره تمرین مقاومتی با شدت کم (40% تا 60% قدرت بیشینه)، تمرین مقاومتی با شدت زیاد (70% تا 90% قدرت بیشینه) و گروه کنترل قرار گرفتند. پروتکل تمرین به مدت هشت هفته سه جلسه‌ای اجرا شد. متغیرها با استفاده از اکوکاردیوگرافی یک و دو بعدی در وضعیت استراحت و فرمول‌های مخصوص اندازه‌گیری شدند. بحث و نتیجه‌گیری: تمرین مقاومتی با شدت کم نسبت به تمرین مقاومتی با شدت زیاد در پسران نوجوانان سالم منجر به برخی تغییرات در ساختار و عملکرد بطن چپ شد، احتمالاً این تغییرات با افزایش بارحجمی و بار فشاری مرتبط می‌باشند. یافته‌ها: در پس‌آزمون نسبت به پیش‌آزمون، در گروه تمرین مقاومتی با شدت کم مقادیر میانگین‌های ضخامت دیوارۀ خلفی بطن چپ در پایان سیستول (028/0 = P) و ضربان قلب استراحت (017/0 = P) به ترتیب افزایش و کاهش معنی‌داری نشان داد. در پس‌آزمون، مقادیر میانگین‌های ضخامت دیوارۀ خلفی بطن چپ در پایان سیستول و شاخص توده بطن چپ در گروه تمرین مقاومتی با شدت کم نسبت به گروه‌ تمرین مقاومتی با شدت زیاد (به ترتیب، 007/0 = P و 005/0 = P) و گروه کنترل (به ترتیب، 005/0 = P و 015/0 = P) و مقدار میانگین توده بطن چپ در گروه تمرین مقاومتی با شدت کم نسبت به گروه تمرین مقاومتی با شدت زیاد (007/0 = P) افزایش معنی‌داری نشان داد.

Title: شناسایی کلبسیلا پنومونیه تولید‌کننده بتالاکتامازهای وسیع‌الطیف KPC در نمونه‌های بالینی در ایران

http://yafte.lums.ac.ir/article-1-1194-fa.html

مقدمه: امروزه کلبسیلا پنومونیه مقاوم به چند دارو تولیدکننده بتالاکتامازها، یکی از عوامل مهم عفونت‌های بیمارستانی محسوب شده و مشکلات درمانی در دنیا ایجاد نموده‌اند. هدف از این مطالعه شناسایی کلبسیلا پنومونیه تولید‌کننده بتالاکتامازهای وسیع‌الطیف KPC (blakpc ) در نمونه‌های بالینی در ایران می‌باشد. مواد و روش‌ها: پس از تعیین هویت تا سطح گونه با استفاده از روش‌های کشت و بیوشیمیائی، تعیین حساسیت 180 ایزوله کلبسیلا پنومونیه به 14 آنتی‌بیوتیک مختلف با استفاده از روش دیسک دیفیوژن انجام گردید و حداقل غلظت بازدارندگی (MIC) برای مروپنم و ایمیپنم تعیین شد. سپس تمامی ایزوله‌های کلبسیلا برای وجود ژن blakpc با روش مولکولی PCR مورد بررسی قرار گرفتند. بحث و نتیجه‌گیری: با توجه به وجود ژن blakpc در کلبسیلا پنومونیه و امکان انتقال افقی این ژنها به باکتری‌های دیگر‌، بایستی ضمن تغییر در الگوی مصرف آنتی بیوتیک‌ها، به معیارهای کنترل عفونت‌های بیمارستانی توجه بیشتری گردد. یافته‌ها: در این تحقیق از 202 ایزوله کلبسیلا، 180 ایزوله (1/89%) به عنوان کلبسیلا پنومونیه و 22 ایزوله به عنوان کلبسیلا اکسی‌توکا (9/10%) تعیین هویت شدند که بیش از 55% کلبسیلا پنومونیه‌ها مقاوم به چند دارو بودند و به مروپنم و ایمیپنم مقاومت بالای 40% نشان دادند. MIC ایزوله‌های مقاوم به کرباپنم‌ها بالای µg/ml 32 بود. نتایج PCR نشان داد که 22 مورد (9/11%) از ایزوله‌ها دارای ژن blakpc بودند که اغلب این ایزوله‌ها از نمونه‌های ادرار و خون بیماران بستری در ICUو بخش اطفال جداسازی شده بودند.
